# Research progress on the mechanism of traditional Chinese medicine regulating intestinal microbiota to combat influenza a virus infection

**DOI:** 10.1186/s12985-023-02228-3

**Published:** 2023-11-13

**Authors:** LanYing Ma, Lingyun Ji, Tong Wang, Zhe Zhai, PeiWei Su, YaNan Zhang, Yuan Wang, WenXiao Zhao, ZhiChun Wu, HuaYun Yu, HaiJun Zhao

**Affiliations:** 1 College of Traditional Chinese Medicine, Shandong University of Traditional Chinese Medicine, Jinan, 250355 Shangdong Province China; 2 School of Nursing, Shandong University of Traditional Chinese Medicine, Jinan, China; 3 Shandong Co-innovation Center of Classic Traditional Chinese Medicine Formula, Shandong University of Traditional Chinese Medicine, Jinan, China; 4 First Clinical Medical College, Shandong University of Traditional Chinese Medicine, Jinan, China

**Keywords:** Influenza a virus, Gut microbiota, Traditional Chinese medicine, And respiratory virus Infections

## Abstract

Influenza A viruses (IAV) are a prevalent respiratory pathogen that can cause seasonal flu and global pandemics, posing a significant global public health threat. Emerging research suggests that IAV infections may disrupt the balance of gut microbiota, while gut dysbiosis can affect disease progression in IAV patients. Therefore, restoring gut microbiota balance may represent a promising therapeutic target for IAV infections. Traditional Chinese medicine, with its ability to regulate gut microbiota, offers significant potential in preventing and treating IAV. This article provides a comprehensive review of the relationship between IAV and gut microbiota, highlighting the impact of gut microbiota on IAV infections. It also explores the mechanisms and role of traditional Chinese medicine in regulating gut microbiota for the prevention and treatment of IAV, presenting novel research avenues for traditional Chinese medicine-based IAV treatments.

## Background

Influenza A viruses (Influenza A viruses, IAV) are a frequent respiratory pathogen that can cause seasonal flu and global pandemics, with clinical symptoms that include fever, cough, chills, and sweating [1]. Seasonal flu outbreaks result in severe cases ranging from 3 to 5 million and 300,000-500,000 deaths worldwide each year [2–5]. However, The emergence and global spread of the H1N1 influenza pandemic in 2009, as well as the recent cases of fatalities caused by H5N1 and H7N9 influenza viruses, highlight the limitations of current strategies for preventing and controlling influenza A infections [6]. Currently, the prevention and treatment of influenza A virus mainly rely on influenza vaccines and antiviral drugs such as neuraminidase inhibitors (oseltamivir and zanamivir) and M2 ion channel blockers [7, 8]. However, the widespread use of these antiviral drugs can lead to drug resistance and adverse reactions [9, 10]. Moreover, due to the high variability of IAV, the current influenza vaccine loses its effectiveness due to antigenic drift and shift of the viral surface antigen, resulting in prevention and treatment failure [11]. Therefore, there may be a close relationship between gut microbiota and the prognosis of IAV infection.

Mounting evidence suggests that the intestinal microbiota of patients with IAV undergoes significant changes. Notably, one study revealed that the intestinal microbial community of H1N1 patients exhibited reduced diversity and relative abundance of beneficial microbes compared to those of healthy controls [12]. Similarly, another study identified intestinal microbial dysbiosis in H1N1 patients, marked by depletion of fungi such as *Aspergillus* and *Penicillium*, and enrichment of Candida. The study also found a correlation between *Aspergillus-induced* increase in C-reactive protein levels and *mycobiota-induced* decrease in calcitonin levels, leading to clinical symptoms [13]. Overall, the composition of the intestinal microbiota in IAV patients is significantly altered compared to non-IAV patients, and these changes may have important implications for clinical symptoms. Therefore, the relationship between intestinal microbiota and IAV infections holds substantial clinical importance.

Traditional Chinese Medicine (Traditional Chinese Medicine, TCM) has a long history of use in China for treating respiratory diseases and recent research has demonstrated its effectiveness in treating the 2019 novel coronavirus, SARS coronavirus, MERS coronavirus, H7N9 avian influenza virus, and H1N1 influenza virus [14–16]. Research has also shown that Chinese herbal medicine can regulate the composition and function of gut microbiota, while the gut microbiota can transform and absorb the components of these medicines [17, 18]. For instance, studies have revealed that Ephedra polysaccharides can increase the relative abundance of *rod-shaped bacteria*, *lactobacilli*, *Prevotella*, *clostridia*, and *Veillonella* while reducing the relative abundance of *Bacteroides* and *Ruminococcus* in the gut of mice with asthma-like airway inflammation. This results in higher production of acetic, propionic, butyric, isobutyric, valeric, isovaleric, and hexanoic acids, reduced eosinophil cells in bronchoalveolar lavage fluid (bronchoalveolar lavage fluid, BALF), as well as decreased serum Immunoglobulin E (Immunoglobulin E, IgE), Interleukin-6 (Interleukin-6, IL-6), Tumor Necrosis Factor α (Tumor Necrosis Factor α, TNF-α), and Interleukin-1β (Interleukin-1β, IL-1β) levels [19]. Although oral ginsenoside utilization rates are low and have poor activity, *bifidobacteria*, *clostridia*, and *rod-shaped* bacteria can transform them into more active ginsenoside compounds [20]. In conclusion, TCM has significant therapeutic effects in regulating the gut microbiota and has tremendous potential in the prevention and treatment of IAV. This article presents a comprehensive summary of relevant research, elucidating the relationship between IAV and gut microbiota, the advantages and effectiveness of TCM in preventing and treating IAV, and the mechanisms by which TCM regulates the gut microbiota to prevent and treat IAV. It provides new strategies and targets for the prevention and treatment of IAV.

## The role of gut microbiota and its relationship with influenza a virus infection

The human microbiota is a complex microbial system that coexists with humans, and the microbiota residing in the gut is composed of approximately 10^14^ known bacteria [21], including *Firmicutes*, *Bacteroidetes*, *Proteobacteria*, *Verrumicribia*, *Actinobacteria*, *Cyanobacteria*, among others. The gut microbiota plays a vital role in maintaining intestinal mucosal integrity, regulating immune and nervous systems, synthesizing nutrients and metabolites, and defending against harmful pathogens [22–24]. Research has shown that alterations in gut microbiota composition can lead to increased susceptibility to respiratory acute infections and chronic lung diseases [25, 26]. Conversely, modifications in respiratory microbiota can affect gut microbiota through the bloodstream [27–29]. In critically ill patients with respiratory infections, genome-wide analysis revealed a correlation between the microbial communities present in the lung and gut [30]. Further investigations by Stefanie Gauguet and others [31] showed that mice lacking *segmented filamentous bacteria* (segmented filamentous bacteria, SFB) had increased susceptibility to lung inflammation and higher mortality rates than mice colonized with SFB. However, after colonization with SFB, even in SFB-lacking mice, the Th17 immune response increased, and levels of IL-22 in BALF, as well as the number of T cell receptor β (T cell receptor β, TCRβ) cells and neutrophils, increased, indicating that gut microbiota can modulate the lung immune response and alleviate severe lung infections.

There is an established relationship between IAV infection and the gut microbiota. IAV infection can alter the composition and metabolites of the gut microbiota, leading to changes in gut barrier function and immune responses. This can result in secondary respiratory infections and impact disease prognosis. A study conducted by Groves et al. [32] demonstrated a significant change in the richness and diversity of the gut microbiota in response to IAV infection, including an increase in the number of bacteria in the *phylum Bacteroidetes* and a decrease in the number of bacteria in the *phylum Firmicutes*. Likewise, another study revealed that influenza infection alters the gut microbiota, promoting the consumption of gut-specific *anaerobic bacteria* and the enrichment of *Bacillus deformis*, leading to gut dysbiosis [33]. Furthermore, proteomic analysis has shown that influenza virus infection reduces the abundance of *Helicobacteraceae and Clostridiaceae*, leading to decreased protein synthesis of short-chain fatty acids and affecting the functionality of the gut microbiota [34]. Conversely, a mouse model where the gut microbiota was reduced demonstrated increased bacterial translocation, inflammation, organ damage, and mortality [35]. Positive correlations have been found between the changes in C-X-C motif chemokine ligand 1 (C-X-C motif chemokine ligand 1, CXCL1), CC chemokine ligand 2 (C-C chemokine ligand 2, CCL2), and the abundance of *Bacteroides*, *Parabacteroides*, and *Alistipes*, while negative correlations were observed between the changes in CC chemokine ligand 3 (CC chemokine ligand 3, CCL3), CC chemokine ligand 5 (CC chemokine ligand 5, CCL5), and the abundance of *Prevotella* and *Butyrivibrio*. Additionally, the abundance of *Proteus* was found to be directly proportional to the weight changes in infected mice [36]. Furthermore, dysbiosis of the gut microbiota induced by IAV infection not only leads to bacterial overgrowth in the gut but also creates favorable conditions for bacteria that are originally present in the respiratory tract, which ultimately weakens the defense threshold against invading pathogens in the lung [37]. Valentin Sencio et al. [38] found that gut dysbiosis in mice under IAV infection conditions impaired their resistance to Streptococcus pneumoniae infection, ultimately increasing the incidence of bacterial recurrence in the lung. These studies demonstrate that the gut microbiota and IAV infection have a reciprocal effect on each other and significantly impact disease development and prognosis after IAV infection.

## The therapeutic effect of traditional Chinese medicine in regulating intestinal microbiota against influenza a virus infection

Modern pharmacological research has demonstrated that traditional Chinese medicine has numerous advantages due to its multi-component and multi-target efficacy, enabling it to effectively improve clinical symptoms such as fever, cough, and respiratory failure caused by viral infections, and reduce pathological changes [16]. For example, the Shuang Huanglian preparation is a novel traditional Chinese medicine formulation composed of *Radix Scutellariae, Forsythia Fructus*, and *Flos Lonicerae*. It possesses the functions of dispelling wind, clearing heat, and detoxification. Animal experiments have demonstrated its potential in reducing the lung index of H1N1-infected mice, downregulating the expression of inflammatory factors(TNF-α, IL-1β, IL-6), and inhibiting the release of IFN-β in bronchoalveolar lavage fluid (BALF). Additionally, it significantly improves the survival rate and extends the lifespan of infected mice [39]. Additionally, Compound Honeysuckle Granules(The composition consists of *Artemisia annua L.*, *Lonicerae japonica Thunb.*, *Schizonepeta tenuifolia (Benth.) Briq.*, *Mentha haplocalyx Briq.*, *Chrysanthemum indicum L.*, *Isatidis tinctorial L.*, *Forsythia suspensa (Thunb.) Vahl.*, *Commelina communis L., Peucedanum praeruptorum Dunn*, and *Glycine max (L.) Merr*)have demonstrated broad-spectrum anti-influenza virus activity in vitro and significant protective effects against lethal influenza virus infection in mice, improving their survival rate, disease symptoms, blood parameters, lung index, and pulmonary pathological changes [40]. Furthermore, researchers have found that traditional Chinese medicine’s significant therapeutic effect on the H1N1 influenza virus is closely related to intestinal flora. Jinzheng Oral Liquid is composed of *Bovis Calculus Atifactus, Rhei Radix et Rhizoma, Caprae Hircus Cornu, Gypsum Fibrosum, Scutellariae Radix, Fritillariae Ussuriensis Bulbus, Chloriti Lapis*, and *Glycyrrhizae Radix et Rhizoma*. It possesses the effects of clearing heat and detoxification, eliminating phlegm, and relieving cough. Research has shown that it can alleviate the imbalance of intestinal flora caused by H1N1 infection in mice by regulating the content of *lactobacillus*, thus reducing pulmonary inflammation and edema in influenza-infected mice [41]. Similarly, The traditional formula Qingfei drink, composed of *Ephedra sinica Stapf, Prunus armeniaca L., Lycopodiella cernua (L.) Pic. Serm., Scutellaria baicalensis Georgi, Bombyx batryticatus, Reynoutria japonica Houtt., Scleromitrion diffusum (Willd.)R.J. Wang, Houttuynia cordata Thunb., Trichosanthes kirilowii Maxim, Platycodon grandiflorus (Jacq.) A.DC.,Forsythia suspensa (Thunb.) Vahl, and Glycyrrhiza glabra L.*, has been found to have the effect of clearing the lungs and relieving asthma. Experimental studies have shown that it can alleviate lung damage, improve survival rate, and reduce lung viral load in mice infected with influenza. Furthermore, studies have revealed that Qingfei drink can enhance the relative abundance of fecal bacteria, such as *Enterococcus*, *Ruminococcus*, *Lactobacillus*, and *Prevotella*, in infected mice. It also alleviates the reduction in the abundance of *Escherichia coli*, *Proteus*, *Acinetobacter*, and *Bacteroides* [42]. Intervention with Andrographis paniculata (including flavonoids and polysaccharides from Houttuynia cordata) has been shown to significantly increase the survival rate of H1N1-infected mice, prolong their lifespan, reduce their lung index, and decrease the production of inflammatory cells. Additionally, it has been found to regulate intestinal flora, and reduce the proportion of *pathogenic Proteus mirabilis*, and cytokine secretion. This has a synergistic effect in reducing lung and intestinal injuries while significantly improving intestinal mucosal barrier function [43].

To summarize, TCM has a significant therapeutic effect on H1N1 influenza virus infection, with its mechanism of action being closely related to the regulation of intestinal flora.

**Fig. 1:**
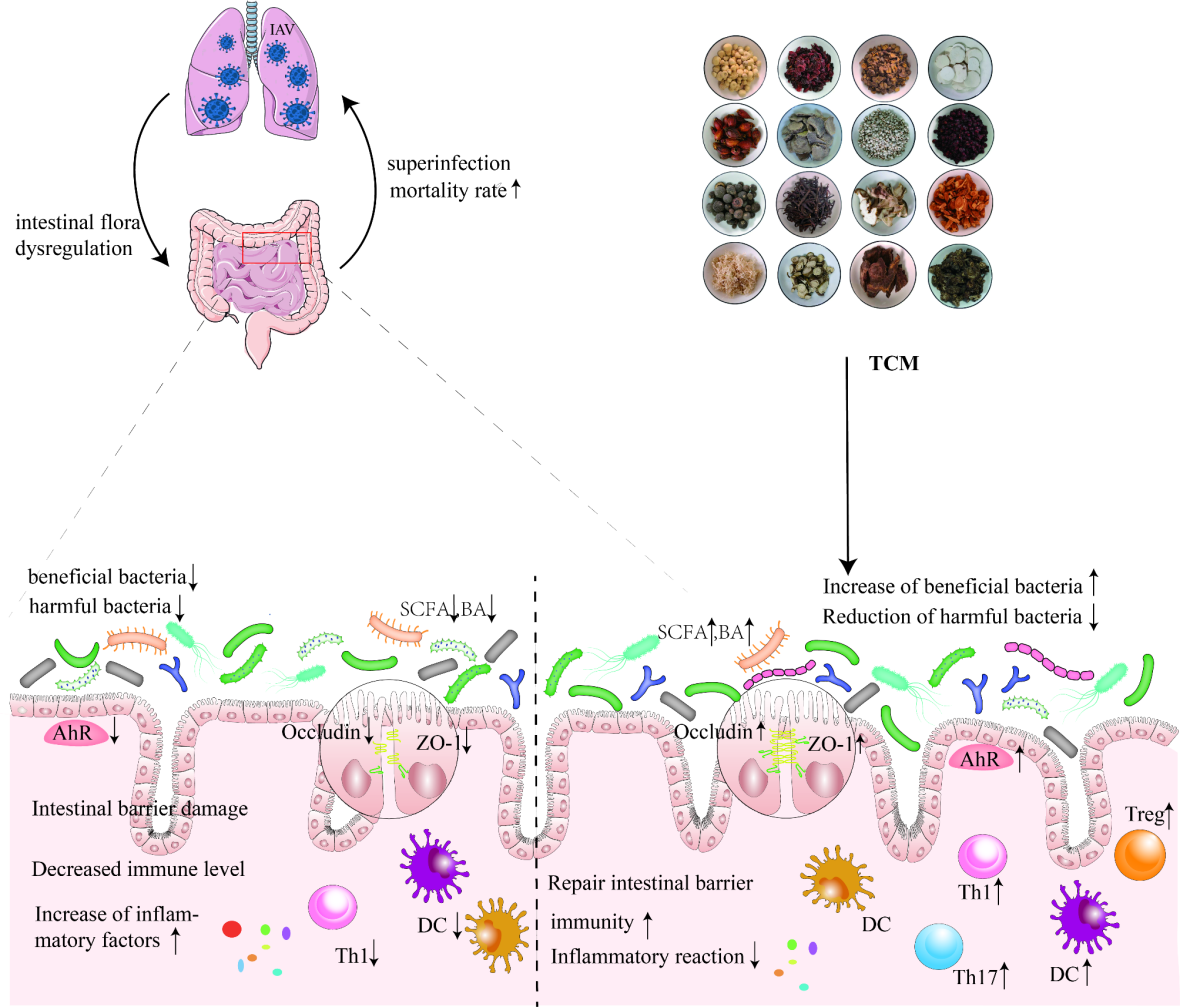
After infecting the lungs, IAV can alter the composition and metabolism of the gut microbiota, leading to compromised intestinal mucosal barrier, impaired immune function, and elevated levels of inflammatory factors. Nonetheless, intervention with traditional Chinese medicine has the potential to modulate the gut microbiota, restore homeostasis, preserve the integrity of the intestinal mucosal barrier, enhance immune function, and regulate inflammatory responses

## The mechanism by which traditional Chinese medicine regulates gut microbiota to prevent and treat IAV

### Regulating the composition and homeostasis of gut microbiota

The stability and composition of the gut microbiota are closely linked to the prognosis of influenza A virus infection. It has been demonstrated that alterations in the gut microbiota post-infection can impact host immune responses, heighten susceptibility to recurring infections, and aid pathogen growth - all of which can ultimately contribute to exacerbating lung inflammation and worsening disease [44]. Studies into the efficacy of TCM for treating influenza A virus have found that its mechanism is intricately tied to regulating the composition and stability of the gut microbiota. For instance, Xuanbai Chengqi Decoction (XBCQ) is composed of *Gypsum Fibrosum, Radix et Rhizoma Rhei, Percarpium Trichosanthis Kirlowiis*, and *Semen Pruni Armeniacae*. It has the functions of clearing heat and phlegm from the lungs, purging heat from the intestines. Animal experiments have shown that XBCQ can significantly improve the survival rate of mice infected with IAV and alleviate lung and intestinal damage in mice. The mechanism of action is related to the reduction in the relative abundance of *Enterobacteriaceae* and *Bacteroidaceae* and the increase in the relative abundance of *Firmicutes* and *Lachnospiraceae* [45]. Likewise, Ephedra polysaccharide can improve acute lung injury caused by influenza by substantially increasing the abundance of *Lactobacillaceae* and *Bifidobacteriaceae* in the gut microbiota [46]. Furthermore, research by Liu et al. [47] has demonstrated that the oral administration of Cangma Huadu granules (CMHD)(The herbal formula is composed of *roasted Atractylodes macrocephala, Ephedra sinica, Agastache rugosa, Sophora flavescens, Eupatorium fortunei, Belamcanda chinensis, Physalis angulata*, and *Glycyrrhiza uralensis*. It possesses the functions of dampness elimination, spleen invigoration, heat clearance, and detoxification) to mice infected with influenza can reduce mortality, alleviate weight loss, lower lung virus titers, and alleviate lung pathological injury. CMHD has also been found to decrease the levels of IL-1β and TNF-α while increasing the levels of IL-10, superoxide dismutase, and glutathione peroxidase. The underlying mechanism may involve CMHD’s ability to regulate the abundance of certain gut microbes, including *Bifidobacterium*, *Clostridium*, *Bacteroides*, and *Prevotella*, in mice. Similarly, polysaccharides found in fish-mint have been found to reduce mucus production and regulate gut microbiota composition in mice infected with influenza, resulting in decreased expression of hypoxia-inducible factors and reduced levels of Toll-like receptors and IL-1β, and increased production of Interleukin-10 (Interleukin-10, IL-10) [48]. Additionally, The Lianhua Qingwen Capsule (LHQW) consists of *Forsythiae Fructus, Lonicerae Japonicae Flos, Ephedrae Herba, Armeniacae Semen Amarum, Gypsum Fibrosum, Isatidis Radix, Asteris Radix et Rhizoma, Houttuyniae Herba, Pogostemonis Herba, Rhei Radix et Rhizoma, Rhodiola Crenulata, Menthae Haplocalycis Herba* and *Glycyrrhizae Radix*. It possesses the functions of clearing away heat and toxins, promoting lung function, and experimental studies have demonstrated that it can reduce virus load in the lungs of influenza-infected mice. Additionally, it can alleviate weight loss and prolong survival time in infected mice, mitigate damage to lung and intestinal mucosa barriers, reverse the decrease in alpha diversity of gut microbiota, and significantly increase the abundance of *Bacteroidetes* and *Muribaculaceae*. The mechanism of action is related to LHQW’s ability to restore gut microbiota homeostasis, repair intestinal mucosa, and regulate the TLR4/NF-κB signaling pathway in the lungs [49]. In summary, TCM has been demonstrated to reduce the inflammatory response in the lungs caused by influenza A virus by regulating gut microbiota composition and stability (as shown in Fig. 1).

### Regulating the function of the intestinal mucosal barrier

The dysfunction of the intestinal mucosal barrier function after infection with the influenza A virus is closely related to the severity of the infection. Studies have shown that the intestinal mucosal barrier is constructed by intestinal epithelial cells, which secrete various immune factors and transmit bacterial antigens, playing an important role in maintaining the symbiotic relationship between the intestinal flora and the host [50], and acting as a control switch for maintaining the stability and ecological balance of the intestinal flora [51]. Influenza virus infection affects the intestinal barrier by damaging the function of tight junction proteins and adhesion proteins in intestinal epithelial cells, which regulate intestinal barrier function and prevent large molecules (such as bacteria and toxins) in the intestinal cavity from entering the bloodstream. Influenza virus infection affects the intestinal barrier by damaging the function of tight junction proteins and adhesion proteins in intestinal epithelial cells, which regulate intestinal barrier function and prevent large molecules (such as bacteria and toxins) in the intestinal cavity from entering the bloodstream. A decrease in the expression of these proteins can lead to impaired barrier function, thereby increasing the disease progression of influenza A virus and the occurrence of critical illness [52]. The study found that H1N1 infection in mice caused a significant decrease in the expression of tight junction proteins in both the lungs and colon. This reduction led to damage to the barrier structure in these organs [53], allowing for the translocation of intestinal bacteria. Subsequently, secondary bacterial infections occurred via fluid circulation [54]. Multiple studies have shown that TCM treatment of influenza A virus is related to the regulation of the intestinal mucosal barrier. Zhu et al. [55] discovered that Houttuynia cordata Polysaccharide can improve the survival rate of H1N1-infected mice, protect their lungs and intestines from damage, and reduce virus replication. The mechanism behind this improvement is that HCP significantly reduces the concentration of pro-inflammatory cytokines/chemokines in the lungs and the number of intestinal goblet cells while increasing physical and immune barriers in the intestines by raising the levels of intestinal secretory immunoglobulin A (secretory immunoglobulin A, sIgA) and tight junction protein zonula occluden-1(zonula occluden-1, ZO-1). Similarly, Prim-O-glucosylcimifugin (Prim-O-glucosylcimifugin, POG), an extract of TCM Saposhnikovia divaricate, has been shown to adjust the structure of intestinal flora and repair the intestinal immune barrier by upregulating the expression levels of tight junction proteins Occludin, Claudin-3, and ZO-1 [56]. Furthermore, 999 XiaoErGanMao granules(The composition consists of *Pogostemon cablin Benth., Chrysanthemum morifolium Ramat., Forsythia suspensa Vahl, Isatis indigotica Fort., Isatis tinctoria L., Rehmannia glutinosa Libosch., Cortex Lycii, Cynanchum atratum Bge., Mentha canadensis Linnaeus*, and *Gypsum Fibrosuum*), a cold granule for children, can relieve weight loss in H1N1-infected mice, reduce the levels of inflammatory cytokines such as IL-6 and IL-1β, decrease lung index and pathological damage, protect the intestinal barrier by maintaining the number of colon goblet cells, and reduce the expression of interleukin 17 A (interleukin 17 A, IL-17 A) in colon tissue [57]. Likewise, Cui et al. [58] found that anthraquinone-glycoside preparations in Rheum palmatum not only increase the abundance of some probiotics and short-chain fatty acid (SCFA)-producing bacteria in the rat intestine but also enhance intestinal barrier function by upregulating the expression levels of ZO-1 and occludin, thereby inhibiting inflammation. As such, the regulation of the intestinal mucosal barrier by TCM to maintain the stability of the intestinal flora and alleviate the infection of influenza A virus is an important mechanism for its action (as shown in Fig. 1).

### Regulating the composition of gut microbiota can have an impact on immune function and inflammatory response

The gut microbiota plays a critical role in regulating the severity of IAV infection by modulating host immunity [59]. Studies have shown that manipulating the gut microbiota to influence both innate and adaptive immunity is an effective approach to combating viral infections [60]. Following influenza virus infection, CCR9^+^ CD4^+^ T cells, which are effector cells derived from the lung, are recruited to the small intestine where they secrete Interferon-γ(Interferon-γ, IFN-γ). This leads to an imbalance in the gut microbiota that promotes Th17 cell polarization in the small intestine. Ultimately, this results in IL-17 A secretion, which mediates immune damage [61]. Numerous studies have demonstrated that traditional Chinese medicine can have antiviral effects by regulating the gut microbiota and modulating host immunity. For example, GeGen QinLian decoction (GeGen QinLian decoction, GQD) composed of *Scutellariae Radix, Coptidis Rhizoma, Puerariae Lobatae Radix*, and *Glycyrrhizae Radix et Rhizoma*, exhibits the effects of relieving muscle tension, clearing heat, and stopping diarrhea. Studies have shown that the treatment with GQD can increase *Akkermansia_muciniphila, Desulfovibrio_C21_c20*, and *Lactobacillus_salivarius* in the intestines of mice infected with influenza A virus, while reducing *Escherichia coli*. This leads to a decrease in the mortality rate and improved lung inflammation in influenza-infected mice. Furthermore, the combination of GQD with fecal microbiota transplantation can suppress the inflammatory differentiation of CD4 + T cells and exhibit systemic protection. These findings suggest that GQD can influence systemic immunity by modulating the gut microbiota [62]. The Feixi Tiaozhi Fang (FTF) is composed of *Astragalus membranaceus, Saposhnikovia divaricata, Angelica dahurica, Ardisia crenata, Magnolia biondii, Prunus armeniaca, Lepidium apetalum* and *Glycyrrhiza uralensis*. Liu et al. [63] have shown that FTF can increase Desulfovibrio in the gut microbiota of rats, while decreasing *Ralstonia and Blautia* in the lung microbiota. FTF has been found to significantly elevate the levels of sIgA and SCFAs in lung and intestinal tissues, indicating its ability to regulate the composition and structure of the lung and gut microbiota, as well as the levels of sIgA in the lung and gut. Moreover, a correlation analysis between the gut microbiota and sIgA in rats revealed a negative correlation between *g__Lactobacillus* and gut mucosal sIgA, suggesting that *g__Lactobacillus* may inhibit intestinal mucosal immunity. Xuanfei Baidu decoction(The composition consists of *Ephedrae Herba, Polygoni Cuspidati Rhizoma et Radix, Glycyrrhizae Radix et Rhizoma, Coicis Semen, Gypsum Fibrosum, Atractylodis Rhizoma, Artemisia Annua Herba, Pogostemonis Herba, Descurainiae Semen Lepidii Semen, Verbenae Herba, Phragmitis Rhizoma, Exocarpium*, and *Armeniacae Semen Amarum*) has been found to regulate gut microbiota diversity and positively correlate with the changes in *Bacteroides, Escherichia-Shigella, Eubacterium nodatum, Turicibacter*, and *Clostridium sensu stricto 1*, which are associated with TNF-a levels. Additionally, Xuanlung Baidu decoction can reconstruct gut immunity by down-regulating the Th1/Th2 ratio [64]. Rosin acid has been shown to regulate the relative abundance of inflammatory bacteria in the gut microbiota of mice by reducing the levels of Anaerotruncus and *Christensenella*, and increasing the levels of *Kurthia, Citrobacter*, and *Klebsiella*. Moreover, it can modulate the Th17/Treg balance in the spleen of mice and down-regulate serum TNF-α and IL-17 A levels [65]. Finally, the inflammation-nourishing syrup, a traditional Chinese medicine formulation, has been demonstrated to improve the inflammatory response by modulating gut microbiota, increasing the production of microbial-derived short-chain fatty acids, and regulating the Th1/Th2 and Treg/Th17 cell balance [66]. These findings highlight the important role of traditional Chinese medicine in regulating gut microbiota to impact immune function and alleviate inflammation in the context of influenza infection (as shown in Fig. 1).

### The metabolic products that regulate the gut microbiota

The changes in intestinal metabolites are closely linked to the development of disease following infection with influenza A virus. The study conducted by Becattini et al. [67] reveals that the metabolic products generated by the gut microbiota can elicit a response to acute host immune activation, thereby influencing the host’s susceptibility and resistance to diseases. For instance, desaminotyrosine (DAT), an intestinal metabolite, can enhance the body’s resistance to the influenza virus by elevating the level of type I interferon (IFN), thereby reducing weight loss and mortality in influenza-infected mice [68]. Similarly, SCFAs including acetate, propionate, and butyrate [69], act not only on the colonized intestine of symbiotic bacteria but also on intestinal immune cells, regulating the immune response via the inflammasome complex [70–72]. It has been observed that SCFA supplements in influenza-infected mice reduce the risks of local and systemic bacterial infections, resulting in reduced pathological changes in the lungs and improved survival rates [38]. Several reports have highlighted the role of traditional Chinese medicine in regulating intestinal metabolites to combat the influenza A virus. For instance, fish mint polysaccharides have been found to regulate Th17/Treg balance by controlling the intestinal flora and the metabolism of fish mint polysaccharide metabolites (acetates) in influenza-infected mice, thereby alleviating the severity of influenza infection [73]. Similarly, Macrocephalae Rhizoma regulates the balance of eight bacterial genera, namely *Akkermansia, Allobaculum, Anaerovorax, coriobacteriaceae_uc-002, Lachnoclostridium, ruminantium_group, tanbacteria*, and *Mur ibaculacea* in rat intestines, thereby maintaining the stable production of SCFAs in the intestines, maintaining intestinal homeostasis, and reducing inflammatory reactions [74]. Xiaoyao powder, composed of *Angelica sinensis, Paeonia lactiflora, Bupleurum chinense, Poria cocos, Atractylodes macrocephala*, and *Glycyrrhiza uralensis*, possesses the effect of soothing the liver and invigorating the spleen. Animal experiments have shown that Xiaoyaosan can reduce the abundance of bacteria that produce short-chain fatty acids (SCFAs), leading to a decrease in SCFA levels and a subsequent reduction in the release of inflammatory factors [75]. It has also been reported that Qingfei Jiedu Granules(The composition consists of *Ephedra sinica Stapf, Gypsum, Bupleurum Chinense DC, Scutellaria baicalensis Georgi, Artemisia carvifolia Buch. - Ham. ex Roxb., Atractylodes lancea (Thunb.), Pogostemon cablin (Blanco) Benth., Verbena officinalis L.* and *Glycyrrhiza uralensis Fisch*) has a significant regulatory effect on T and B lymphocytes and reduces the expression of various pro-inflammatory cytokines, which is instrumental in alleviating influenza A virus infection. The metabolomics and 16 S studies revealed that its mechanism of action is linked to the regulation of intestinal citrate and amino acids metabolism, stability of intestinal flora, and enrichment of beneficial bacteria in the intestine [76]. In summary, regulating the metabolites of intestinal flora is a crucial mechanism for traditional Chinese medicine in alleviating influenza A virus infection (as shown in Fig. 1).

## Summary and prospects

TCM has been utilized clinically for over a thousand years, and recent studies have demonstrated its efficacy in reducing lung inflammation and improving clinical symptoms, shortening treatment duration, and promoting recovery in IAV patients. Current challenges in IAV treatment focus on preventing complications and reducing the number of critical cases. Research indicates that the gut microbiota plays a vital role in IAV infection; IAV-infected patients often experience gut dysbiosis and impaired gut barrier function, making them susceptible to secondary bacterial infections that worsen the disease. Conversely, the gut microbiota can also regulate the immune response systemically and locally, significantly impacting patient prognosis. Therefore, regulating and restoring gut microbiota balance is crucial in treating and preventing IAV infection. TCM positively impacts the prognosis of IAV patients by regulating the gut microbiota’s composition, function, gut mucosal barrier, metabolites, immune, and inflammatory response. However, the specific correspondence between components and microbial communities in this regulatory role is currently lacking in research. Furthermore, due to the multitude of components in traditional Chinese medicine, it is not yet clear whether these components directly regulate the gut microbiota in concert or indirectly regulate them through metabolites. Future studies using high-throughput sequencing techniques such as 16 S rRNA gene sequencing, metagenomics, and metabolomics in IAV disease models and traditional Chinese medicine intervention are needed to identify advantageous microbial communities, their metabolites, and metabolic pathways. Additionally, further experiments involving microbial transplantation using advantageous strains will help elucidate the specific pharmacological and molecular mechanisms by which traditional Chinese medicine regulates the gut microbiota.We firmly believe that with the advancement of scientific technology, research on TCM and the gut microbiota has been gradually deepening. Although the specific mechanisms underlying their interaction are intricate, there is hope for a clearer understanding in the future. We look forward to TCM’s continued advantages in regulating the gut microbiota and demonstrating even more remarkable outcomes. In conclusion, the use of TCM in modulating the gut microbiota for the prevention and treatment of IAV holds significant research significance as a novel therapeutic direction.

## Data Availability

Not applicable.
